# Two Ouncss of Potassæ Nitras Taken in Mistake for Epsom Salts

**Published:** 1846-12

**Authors:** Wm. Gillard

**Affiliations:** Surgeon, Totnes


*Two ouncss of Potassæ Nitras taken in mistake for Epsom Salts.*—By Wm. Gillard, Esq., Surgeon, Totnes. January 14th, I was sent for by Captain C., residing about two miles from me, one of whose grooms had taken two ounces of nitrate of potass. He said about five minutes after having taken it, he felt a burning pain in his stomach which was immediately followed by sickness; he then suspected it to be nitre, as he had that evening brought it home with the packet of Epsom salts. On finding it to be the case, he desired that Captain C. might be told of it, who requested him to take some mustard in hot water, and kept up the sickness until I arrived, which I considered sufficient to empty the stomach. As he still complained of the burning pain in his stomach, I gave him about three drachms of carbonate of magnesia, with half a drachm of tincture of opium in four ounces of water. I then ordered him to take another dose of magnesia after three hours, to be plentifully supplied with diluents during the night, and to take an ounce and a half of castor oil in the morning.

15th. He had rested well during the night; the castor oil had acted freely, and he was quite free from pain. To remain in bed, take some meat-broth, and repeat the oil on the following morning.

16th. Quite comfortable. To get up and have meat.

18th. Thought he had some pain in his back. I ordered him half a grain of opium, and fifteen grains of powdered gum-arabic, to be taken in water three times a day for two days; when he said he was quite well, and returned to his work, and has never since complained. *Prov. Med. and Sur. Jour.*

